# Antiphospholipid Syndrome - A Case Report of Pulmonary Thromboembolism, Followed with Acute Myocardial Infarction in Patient with Systemic Sclerosis

**DOI:** 10.3889/oamjms.2015.114

**Published:** 2015-11-08

**Authors:** Marija Vavlukis, Irina Kotlar, Emilija Chaparoska, Bekim Pocesta, Hristo Pejkov, Marjan Boshev, Sasko Kedev

**Affiliations:** *University Clinic of Cardiology, Faculty of Medicine, Ss Cyril and Methodius University of Skopje, Skopje, Republic of Macedonia*

**Keywords:** systemic sclerosis, antiphospholipid syndrome, protrombotic state, pulmonary thromboembolism, myocardial infarction

## Abstract

**AIM:**

We are presenting an uncommon case of pulmonary embolism, followed with an acute myocardial infarction, in a patient with progressive systemic sclerosis.

**CASE PRESENTATION:**

A female 40 years of age was admitted with signs of pulmonary embolism, confirmed with CT scan, which also reviled a thrombus in the right ventricle. The patient had medical history of systemic sclerosis since the age of 16 years. She suffered an ischemic stroke 6 years ago, but she was not taking any anticoagulant or antithrombotic medications ever since. She received a treatment with thrombolytic therapy, and subsequent UFH, but, on the second day after receiving fibrinolysis, she felt chest pain accompanied with ECG changes consistent for ST-segment elevation myocardial infarction (STEMI). Urgent coronary angiography was undertaken, which reviled cloths causing total occlusion in 4 blood vessels, followed with thromboaspiration, but without successful reperfusion. Several hours later the patient developed rapid deterioration with letal ending. During the very short hospital course, blood sampling reviled presence of antiphospholipid antibodies.

**CONCLUSION:**

The acquired antiphospholipid syndrome is common condition in patients with systemic autoimmune diseases, but relatively rare in patients with systemic sclerosis. Never the less, we have to be aware of it when treating the patients with systemic sclerosis.

## Introduction

The antiphospholipid syndrome also known as Hughes Syndrome is an autoimmune condition which is characterized by the occurrence of venous/arterial thrombosis or of specific pregnancy morbidity, in the presence of antiphospholipid antibodies. There are three primary classes of antibodies associated with the antiphospholipid antibody syndrome: anticardiolipin antibodies, the lupus anticoagulant, and antibodies directed against specific molecules including a molecule known as beta-2-glycoprotein. A patient with the antiphospholipid syndrome must meet at least one of two clinical criteria and at least one laboratory criterion.

The clinical criteria, adapted from Miyakis et al., 2006 [1], include: Vascular thrombosis: one or more episodes of arterial, venous or small vessel thrombosis; and Pregnancy morbidity: at least one unexplained death of a normal appearance fetus, at or beyond the 10th week of gestation; at least one pre-term birth of a neonate of normal appearance before 34 weeks of gestation, because of eclampsia or severe pre-eclampsia, or with signs of placental insufficiency; three or more unexplained consecutive spontaneous miscarriages before 10 weeks of gestation where anatomical, hormonal and chromosomal causes have been excluded.

The laboratory criteria include: Lupus anticoagulant (LA) present in the plasma, on two or more occasions at least 12 weeks apart; Anticardiolipin (aCL) antibody present in serum or plasma, in medium or high titer (i.e. ≥ 40 GPL units or MPL units or ≥ 99th centile), on two or more occasions at least 12 weeks apart; and Anti-b2-glycoprotein I antibody in serum or plasma (in titer ≥ 99th centile), present on two or more occasions, at least 12 weeks apart.

There are two main classifications of the antiphospholipid antibody syndrome. If the patient has an underlying autoimmune disorder, such as systemic lupus erythematosus, the patient is considered to have secondary antiphospholipid antibody syndrome. If the syndrome exists as an independent condition, than it is termed primary antiphospholipid antibody syndrome [1-3].

The aim of this study is to present an uncommon case of pulmonary embolism, followed with an acute myocardial infarction, in a patient with progressive systemic sclerosis.

## Case Presentation

Medical history: A 40 year old woman has been suffering from progressive systemic sclerosis, diagnosed at the age of 16 years. She was not regularly followed up, eider for regular check up’s, eider for medications. Her last control at the Rheumatology Clinic was a year ago, and since then she was on methotrexate (10 mg). She was married, but had never been pregnant. Important information from her previous medical history is the fact that she suffered an ischemic stroke 6 years ago, but a work up for hypercoagulability state was never conducted, and any prophylactic anticoagulant or antithrombotic therapy was not given. One week before the admission at Cardiology Clinic, she was hospitalized at the Rheumatology Clinic because of shortness of breath, cough, fatigue and weakness. On physical examination she was pale, with cyanosis on the both hands. She had pinched nose, taut skin with numerous teleangiectasias with retraction of the lips. On auscultation she had decreased breath sounds, absent in the basal parts of the right lung. Her BP was 120/80 mmHg, and HR = 80/bpm. Laboratory tests: polyglobulia with red blood cells 6.0 × 10^12^/L, Hb 172 g/L and hematocrit 0.57; moderate thrombocytopenia (50-100 × 10^9^/L), in two separate controls, and elevated WBC (>20 × 10^9^/L), with neutrophilic predominance (> 90%). Other biochemical parameters were in the normal range. The chest X-ray detected consolidation of the basal parts of the right lung, accompanied with deteriorated respiratory function (blood gas analyses revealed partial respiratory insufficiency: pH 7.446; pCO_2_ 4.06 kPa; pO_2_ 6.99 kPa; sO_2_ 88%). At that point the patient was treated with corticosteroids, antibiotics, bronchodilators and oxygen therapy. After several days she experienced sudden onset of sharp chest pain, shortness of breath, cough, rapid heartbeat and central cyanosis.

**Figure 1:**
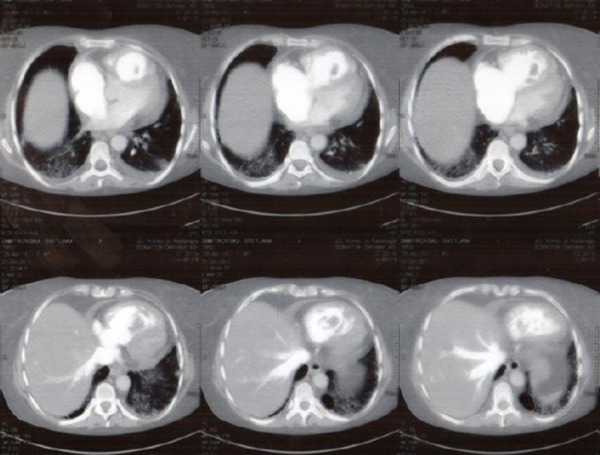
*Cardiac CT showing extensive thrombotic material in right ventricle (RV), small bilateral pleural effusions, and tiny layer of pericardial effusion*.

**Figure 2:**
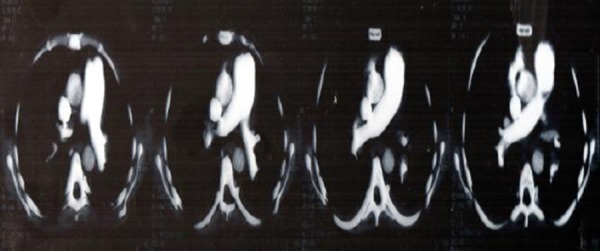
*Cardiac CT of the truncus pulmonalis (TP) and principal branches: dilated TP (40mm), dilated RPA (28mm), thrombotic material in TP, massive occlusive thrombus in RPA, and multiple thrombi in secondary branches*.

Because of a suspicion of pulmonary thromboembolism (PTE), D-dimers were measured, and found to be increased (> 4500 ng/mL), and CT scan with pulmonary embolism protocol was performed. It reviled multiple thrombi in the right ventricle (Figure 1), and massive acute pulmonary embolism involving the right pulmonary artery and extending into the lobar and segmental brunches. Truncus pulmonalis was dilated up to 40 mm in diameter; same was with the right PA, 28mm (Figure 2).

**Figure 3:**
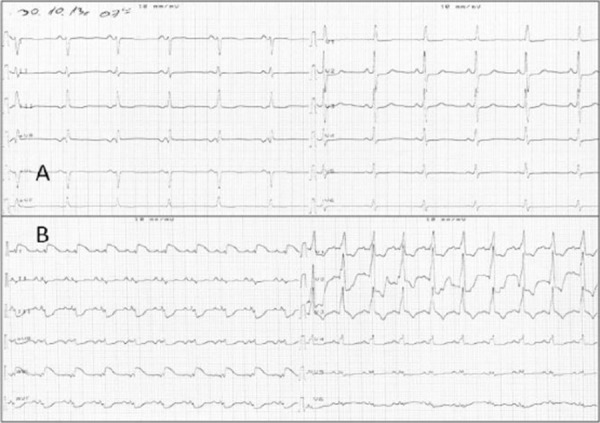
*Patient’s ECG at the moment of diagnosis of BTE: sinus rhythm, 70 bpm, no typical ECG signs for BTE. (A) ECG during sudden chest pain on the second day after she was treated with thrombolytic therapy: sinus tachycardia, 100/bpm, ST-segment elevation in lateral leads: D1 and AVL, RBBB (B)*.

We also registered small bilateral pleural effusion and tiny layer of pericardial effusion (Figure 1). CT scan and transthoracic echocardiography (TTE), indicated dilated right heart cavities, severe tricuspid valve regurgitation, pulmonary hypertension, McConnell’s sign - all together manifestations of right ventricular dysfunction were present, as a result of massive pulmonary thromboembolism, at which point decision for treatment with fibrinolysis was made. There was demonstrating signs of severe illness: was dyspneic, tachypneic, with low sO_2_ saturation (~76% with oxygen mask) and central cyanosis, but hemodynamically stable: HR ~80/bpm, BP 120/80 mmHg. The ECG showed sinus rhythm with signs of right axis deviation and incomplete RBBB (Figure 3A).

**Figure 4:**
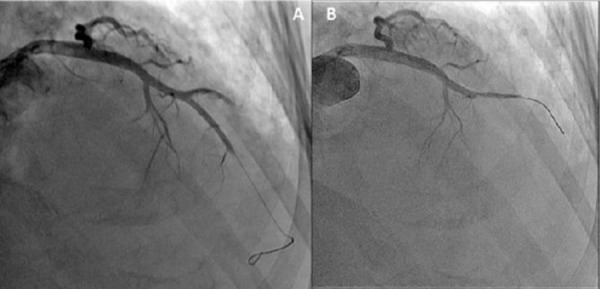
*Coronary angiography, only the LAD (left panel – A), and the diagonal branch (right panel B) are presented. These are post thromboaspiration results (unsuccessful reperfusion)*.

Thrombolytic treatment with streptokinase was applied, using the 24 h administration protocol (250 000 IU during the first hour, followed by 100 000 IU/h in the next 24 hours), with subsequent heparin (UFH) infusion (1000 IU/h). At the very beginning of the UFH infusion, in the morning hours on the second day of the PTE treatment, she again felt chest pain, but now clamping, accompanied with ST elevation in the lateral leads (D1 and AVL), and broad QRS complex (~120 msec), consistent for STEMI (Figure 3B). The patient was immediately sent to the cat lab, where coronarography was performed. The angio study revealed 4 occluded arteries (left anterior descending artery, diagonal branch, circumflex artery and obtuse marginal artery). The only potent one was the right coronary artery. An attempt for thromboaspiration on the left anterior descendent artery and its diagonal branch was undertaken, clot was aspirated from LAD and diagonal brunch, but the TIMI flow remained low (Figure 4).

Despite all the efforts, 20 hours after the intervention, the patient died in cardiogenic shock. Blood sampling for the acquired antiphospholipid syndrome, that we performed, were positive for anticardiolipin (aCL) antibodies. It was the first and only time that suspicion for antiphospholipid syndrome was arisen in this patient with the history for progressive systemic sclerosis.

## Discussion

Today, the cause of the antiphospholipid syndrome is still not well understood. Although the aPL antibodies are clinically linked to APLs, it is not known whether they are involved in pathogenesis, because about 5% of the healthy population, has aPL antibodies. There are several mechanisms which could be responsible for the hypercoagulability state, including: complement activation, production of antibodies against coagulation factors (prothrombin, protein C, protein S), platelet activation, activation of the vascular endothelium, reaction of antibodies to oxidized low density lipoprotein [1-4].

The diagnosis of an antiphospholipid syndrome requires the combination of at least one clinical and one laboratory criterion, but timing of the laboratory test and clinical event is very important. A remote test, avoids false positive results from interference with the event, however, in extreme cases, a positive test separated many years from a clinical manifestation also risks misclassification, as a causative relationship between event and test would then be in doubt. The Sapporo statement encouraged investigators to provide applicable information, but relevant existing data are rather poor. The stability of the laboratory testing over time is reassuring, but spontaneous variation of aPL in individual patients occurs in up to a quarter of cases. Whether disease activity and treatment contribute to assay variability is unknown. The International consensus statement from 2006, suggests that APS should not be classified, if it is more than 5 years between the clinical event and the positive laboratory test, and that an allowance of at least 12 weeks between symptom and test will assist assessment of the relationship between clinical manifestations and aPL. The time limits are valid independently of which feature of APS (clinical or laboratory) occurs first [1].

Except these criteria, adapted from Miyakis, there is a list of other features which are associated with APS but are not included in the revised classification. These include: heart valve disease, livedo reticularis, thrombocytopenia, nephropathy, neurologic involvement, IgA aCL, IgA anti-beta-2-GPI antibodies, antiphosphatidylserine antibodies, antiphosphatidylethanolamine antibodies, antibodies against prothrombin alone, and antibodies to the phosphatidylserine-prothrombin complex [1].

In our patient we had at list three episodes of vascular thrombosis (arterial), including cerebrovascular insult 6 years earlier. We managed to have only one laboratory sampling of APLa, during the actual hospitalization (high positive - 87 GPL units). We were unable to trace any documentation for previous testing, partly because of a lack of patient cooperation and adherence to the medical treatment of the disease. She also had heart valve disease on the mitral valve leaflets, and moderate thrombocytopenia.

We are aware that we don’t fulfil exactly the laboratory criteria, because of the course of the disease we were unable to perform all the necessary laboratory investigations, but according to results of one blood sampling and clinical characteristics we strongly believe that our patient was suffering from an antiphospholipid syndrome secondary to systemic sclerosis, experiencing catastrophic complications.

The connective tissue disorders comprise a number of related conditions that include systemic lupus erythematosus (SLE), the antiphospholipid (Hughes) syndrome, scleroderma, myositis and Sjögren’s syndrome. They are characterized by autoantibody production and other immune-mediated dysfunction. There are common clinical and serological features with some patients having multiple overlapping connective tissue disorders. There are growing evidences about the association of APL syndrome with systemic sclerosis. It is not exclusively associated with systemic lupus or rheumatoid arthritis [5].

In the study conducted by Say and Swetha, published in 2015, the association of antiphospholipid antibodies was found for several connective tissue diseases. 14.7 % of patients with connective tissue disease had positive APLa, mostly in SLE group (73.3% of positive pts.), but also in 13.3% of patients with mixed connective tissue disease (MCTD) and 13.3% of patients with systemic sclerosis. In conclusion they recommend that APL antibodies should be tested in all patients with connective tissue disease [6]. Balanescy and co-workers conducted a study about the association of systemic sclerosis (SSc) and APLa. 38.9% of patients with SSc had antiphospholipid antibody presence associated with low complement level, in the absence of any clinical feature of APLs. Toure, in his study of 40 pts with SSc identified APLa in 57.3% of them [7, 8].

Clinical significance of our finding: The particular role of these antibodies in clinical manifestations of SSc is still unknown [7]. Searching threw the literature; we found a case report on association of APLs with cutaneous systemic sclerosis (SSc), with vascular complications that responded well to oral anticoagulant treatment (OAK) [9]. Also, a complicated case of vascular complication after CABG in patient with SLE, SSc and secondary APLs was referred by Naoto in 2011 [10]. In the study cohort of 72 pts with SSc, 9.7% were positive to APLa, but only one patient had deep vein thrombosis [11]. We were not able to find case reports on SSc associated with APLs, with similar vascular distribution (in arterial bed) as in our patient. Is it only overlapping, or one condition is leading to other, with catastrophic outcome? Would our patient have different outcome if the proper diagnostic workup has been undertaken after the first thrombotic event?

According to the recommendations, the laboratory testing should have been done after the first episode of thrombotic event (the ischemic stroke). The confirmation of the diagnosis of APLs at that point, would mean a proper anticoagulation treatment, which could have prevented the further thrombotic complications and fatal consequences in this patient.

In conclusion, according to our case, and growing body of the literature data, it seems reasonable that patients with SSc should be investigated for APLa, in order to early identify those who have increased risk for worse clinical outcome, and to expose them to more aggressive treatment.
